# Data on the quantitative assessment pulmonary ground-glass opacification from coronary computed tomography angiography datasets

**DOI:** 10.1016/j.dib.2016.10.032

**Published:** 2016-11-05

**Authors:** J. Tobias Kühl, Thomas S. Kristensen, Anna F. Thomsen, Louise Hindsø, Kristoffer L. Hansen, Olav W. Nielsen, Henning Kelbæk, Klaus F. Kofoed

**Affiliations:** a Department of Cardiology, The Heart Centre, Rigshospitalet, University of Copenhagen, Denmark; b Department of Radiology, Diagnostic Centre, Rigshospitalet, University of Copenhagen, Denmark; c Department of Cardiology, Bispebjerg Hospital, University of Copenhagen, Denmark; d Department of Cardiology, Roskilde Sygehus, Roskilde, Denmark

**Keywords:** Cardiac computed tomography angiography, Lung water, Pulmonary congestion

## Abstract

We assessed the CT attenuation density of the pulmonary tissue adjacent to the heart in patients with acute non-ST segment elevation myocardial infarction (J.T. Kuhl, T.S. Kristensen, A.F. Thomsen et al., 2016) [1]. This data was related to the level of ground-glass opacification evaluated by a radiologist, and data on the interobserver variability of semi-automated assessment of pulmonary attenuation density was provided.

**Specifications Table**

**Table:** 

Subject area	*Clinical Medicine*
More specific subject area	*Pulmonary tissue. Lung water. Ground-glass opacification. Interobserver variability*
Type of data	*Image, graph*
How data was acquired	*Computed Tomography angiography.*
Data format	*Analyzed*
Experimental factors	The samples were from patients with acute myocardial infarction before invasive treatment was performed. After sampling data was analyzed using semi-automated commercially available software.
Experimental features	Pulmonary attenuation density was assessed as a measure of lung water. Interobserver variability and relation to patterns of ground-glass opacification.
Data source location	*Copenhagen, Denmark (* 55.6960°N, 12.5666°E)
Data accessibility	Data is with this article

**Value of the data**

• The data can be used to evaluate inter-observer variability of semi-automated assessment of lung water assessed on cardiac computed tomography data sets.

• This data can be used to evaluate the relation between subjective assessment of ground-glass opacification and pulmonary attenuation density.
• These data on high attenuation density volume in patients with myocardial infarction can be used in further studies to compare values with other cohorts.

## 1 Data

The visual severity of three levels of ground-glass opacification (GGO) is related to pulmonary attenuation densities. Further the interobserver variability of these automated measurements are presented.

### 1.1 Interobserver variability

**Fig. 1:**
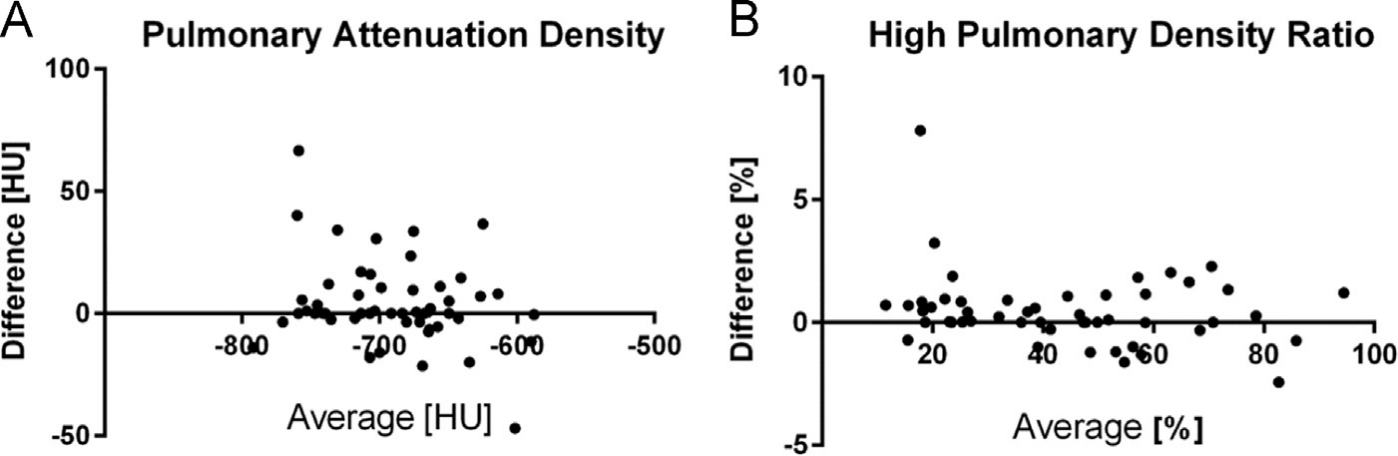
Bland–Altman plot of interobserver variability in the assessment of A: pulmonary attenuation density and B: high pulmonary density ratio.

Automated segmentation of the lungs was performed by two independent observers in 53 patients; 27 with left ventricular ejection fraction ≥45% and 26 patients with left ventricular ejection fraction <45%. No significant difference between observers was found in the measurement of pulmonary attenuation density: mean difference 4 HU (95% CI of −31 to 39 HU). Also no significant difference was found between observers in the measurement of high density ratio (Mean difference of 0.5% with a 95% CI of −2 to 3). Bland–Altman plot of pulmonary attenuation densities and high attenuation volumes are given in Figs. 1A and B.

### 1.2 The subjective level of GGO

**Fig. 2:**
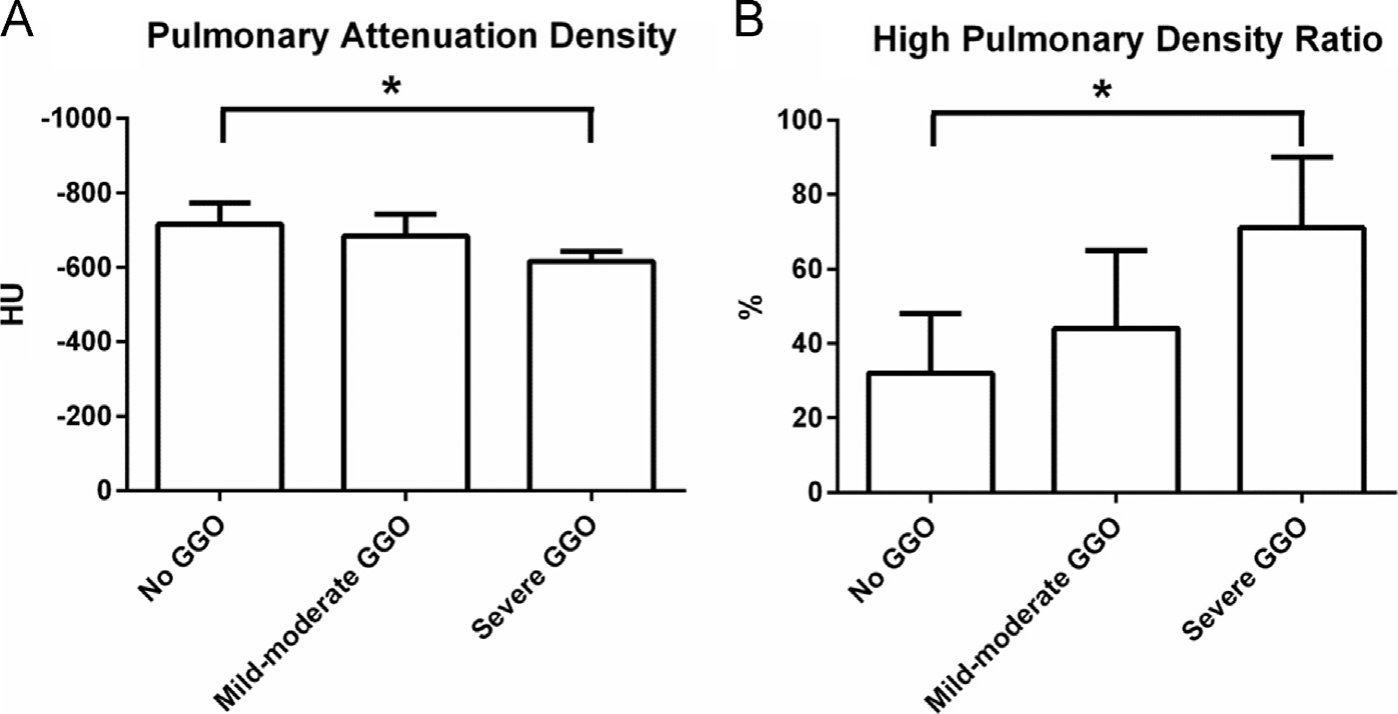
Pulmonary attenuation density [in Hounsfield Units (HU)] and the high pulmonary attenuation density ratio [%], according to the visual assessment of ground-glass opacification (GGO). *p<0.001.

CT data sets from 367 patients with acute non-ST segment elevation myocardial infarction were analysed. No GGO was present in 292 patients mild-moderate GGO was found in 65 patients, and severe GGO in 10 patients. This categorization correlated with increasing mean pulmonary attenuation density and mean high pulmonary density ratio (Figs. 2A and B).

## 2 Experimental design, materials and methods

Coronary computed tomography angiography data sets were acquired before invasive treatment from 367 patients with acute non-ST-segment elevation myocardial infarction [1], [2], [3]. From these datasets the subjective level of GGO was evaluated by an experienced radiologist on a 3 point scale (no GGO, mild-moderate GGO, and severe GGO) to compare with quantitative metrics of lung water (pulmonary attenuation density).

**Fig. 3:**
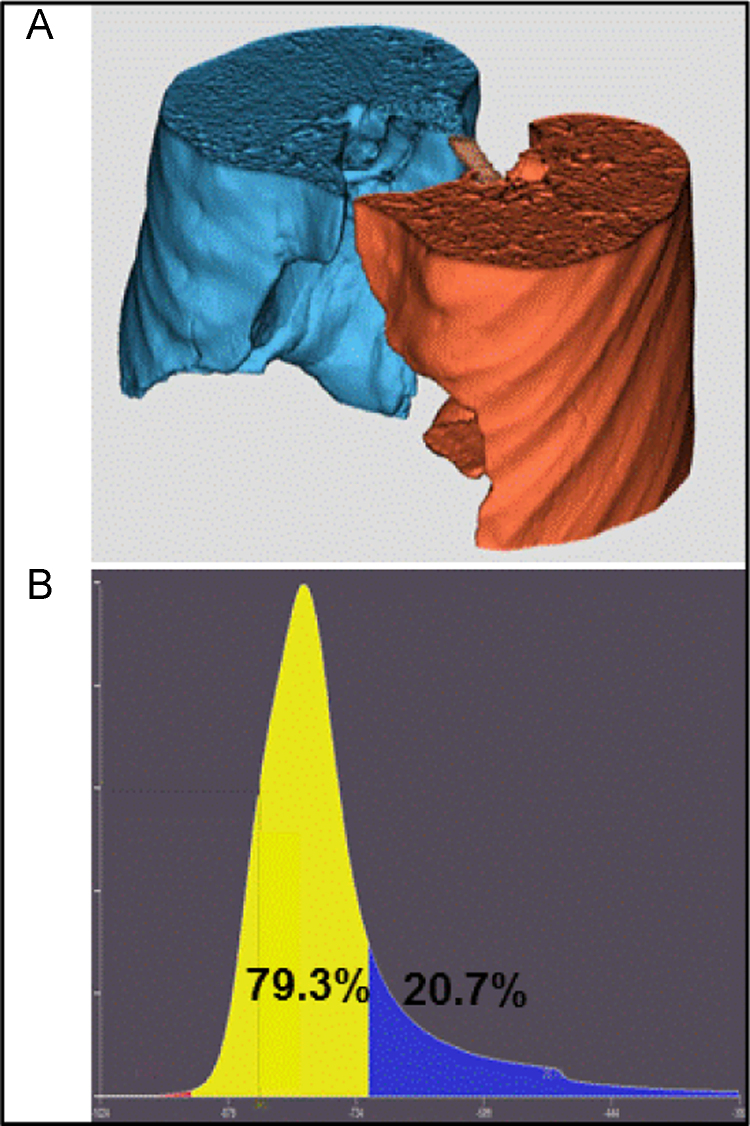
**How to perform quantitative assessment of Ground-glass opacification** (A) The software performs automated segmentation of pulmonary tissue adjacent to the heart. (B) An example of a histogram of the relative frequency of HU values of the automated lung segmentation (excluding all values above −300 HU) shows the percentage of lung tissue with high pulmonary attenuation density > −720 HU (= *high pulmonary density ratio)* in blue color. (For interpretation of the references to color in this figure legend, the reader is referred to the web version of this article).

The pulmonary attenuation density and the high pulmonary density ratio was obtained using pulmonary densitometry in the following way: Full view images in 3 mm slice thickness were loaded into the workstation (Vitrea, version 6.2, Vital, Minnesota) using the pulmonary density analysis tool, which automatically traced the part of the left and right lung within the scan field (Fig. 3A). Voxels with attenuation densities above –300 (water, contrast, and soft tissue) was subtracted from the images in the further analysis. Mean attenuation density and attenuation density histograms were assessed (Fig. 3B). An attenuation density of −720 HU was chosen as a threshold for high pulmonary attenuation density (Three standard deviations above mean from a previous study) [4]. The volume of the lung with attenuation density > −720 defined the *high pulmonary density volume* and the *high pulmonary density ratio* was assessed as: high pulmonary density volume / total measured lung tissue volume, in %.

Comparison between groups of subjective levels of GGO was tested using one way ANOVA. Interobserver variability in the assessment of lung water was evaluated using the Bland–Altman method [5].
